# Care home practitioners’ perceptions of the barriers and facilitators for using off-the-shelf gaming technology with people with dementia

**DOI:** 10.1177/14713012221085229

**Published:** 2022-04-15

**Authors:** Ben Hicks, Anomita Karim, Erin Jones, Malcolm Burgin, Clare Cutler, Wen Tang, Sarah Thomas, Samuel R Nyman

**Affiliations:** Brighton and Sussex Medical School, Centre for Dementia Studies, 12190 University of Sussex, UK; Department of Psychology, 6657 Bournemouth University, Poole, UK; Alive Charity, Bristol, UK; The Wessex Institute, 7423 University of Southampton, UK; Department of Games Technology, 6657 Bournemouth University, Poole, UK; Bournemouth University Clinical Research Unit, 6657 Bournemouth University, Poole, UK

**Keywords:** gaming technology, dementia care, dementia practice, care homes, care staff, digital, qualitative, well-being, theoretical domains framework, the capability, opportunity, motivation and behaviour model

## Abstract

**Background:** Off-the-shelf digital gaming technology has been shown to support the well-being of people with dementia. Yet, to date, it is rarely adopted within dementia care practice, particularly within care homes. Drawing on a descriptive, qualitative approach, this is the first study that has sought to explore care home practitioners’ perceptions of the barriers and facilitators for using gaming technology within their workplace. **Method:** Data were collected across eight focus groups in the south of England with a total of 39 care home workers. These were analysed inductively following the 6-stage thematic process as outlined by Braun and Clarke (2006). **Findings:** Three themes, constructed from the data suggested, the *care environment, staff knowledge and skills for inclusive gaming, and staff perceptions about capabilities (their own and those of people with dementia)* inhibited or facilitated the use of gaming technology in care homes. The findings were interpreted through a combination of the Capability, Opportunity, Motivation and Behaviour model and the Theoretical Domains Framework to provide theory-based insights into the mechanisms for supporting behaviour change and implementation within the care home context. **Conclusions:** We argue for the need to target wider institutional barriers alongside providing inclusive training for care staff on incorporating gaming technology within their person-centred care approaches. Through these mechanisms, they can be provided with the capabilities, opportunities and motivation to integrate gaming technology within their practice, and thus facilitate the process of culture change within care homes.

## Introduction

In recent years the role of technology to support the psychosocial needs of people throughout the dementia care pathway has become widely acknowledged (Kenigsberg et al., 2019; Lorenz et al., 2019). This includes off-the-shelf digital gaming technologies, which are digital platforms such as computers, consoles (e.g. Nintendo Wii and Microsoft Kinect) and tablets (e.g. iPad) that support interactive electronic games for the primary purposes of providing entertainment. Scholars have highlighted how these devices are being incorporated into dementia care practice as a means to provide activities that are stimulating and enjoyable, and have also noted other benefits for people’s well-being (Bowes et al., 2018; Dove and Astell, 2017; Goodall, Taraldsen and Serrano, 2021; Joddrell and Astell, 2016). Specific positive outcomes for people with dementia (evidence to date relates mostly, but not exclusively to community-dwelling people), include opportunities for: promoting cognitive stimulation, mild physical exercise and social interaction; continuing life-long learning; mastering new and sometimes complex skills; and (re)engaging with meaningful and enjoyable activities that can foster self-confidence by challenging people’s perceptions of their own capabilities (Cutler, Hicks and Innes, 2016; Hicks, Innes and Nyman, 2020; Sweeney, Clarke and Wolverson, 2021). Furthermore, the ubiquity of these devices throughout society ensures they are more readily accessible and potentially less stigmatised than dementia-specific technology (Meiland et al., 2017).

Despite these promising findings, further work is required to ensure the widespread adoption of gaming technology within dementia care. This may be particularly pertinent in the current COVID-19 climate, which emphasised the benefits of these devices for mental stimulation and activity as well as enabling people with dementia to remain socially connected whilst adhering to the physical distancing restrictions (Chu et al., 2021; Talbot and Briggs, 2021). To achieve this, researchers have highlighted the need to provide practitioners with evidence-based guidance and training that will enable them to incorporate it within their practice (Hicks, Innes and Nyman, 2020; Weiss et al., 2021).

Care homes are a particularly important arena to consider in this agenda, given the high numbers of residents likely to have dementia and require support to maintain their well-being. In 2014 it was estimated that around 69% of all people living in UK care homes had some form of dementia (Knapp et al., 2014) and in 2016 nearly half of all nursing home residents in the United States (US) were diagnosed with dementia (Harris-Kojetin et al., 2019). However, research continues to demonstrate that residents with dementia experience a lack of opportunities to engage in meaningful activities and socially connect (Chung, 2004; Kolanowski, Fick, Campbell, Litaker, & Boustani, 2009; Tak, Kedia, Tongumpun, & Hong, 2015; Neal, du Toit and Lovarini, 2020). Tak et al. (2015) interviewed 37 residents with dementia from nursing homes in the southern United States. They found that whilst many of them missed previous hobbies, they felt there were limited opportunities and support to engage in activities and many were unmotivated to participate in those on offer. This suggests the need to encourage care home workers (i.e. formal, paid staff whose job role includes providing hands-on care and support to residents living with dementia) to offer more varied and widely appealing activities for people with dementia, and potentially think outside of the stereotypes of those considered ‘dementia-appropriate’ (Genoe, 2010) such as music or life reminiscence initiatives. This is where digital gaming technology initiatives, which offer a vast array of novel games that can be tailored to people’s interests and abilities, may have a role to play.

Before gaming technology can be adopted by care home practitioners it is important to examine the challenges they may encounter when introducing them into their practice. Although the primary focus of care home studies exploring gaming devices has been to evaluate outcomes for people with dementia, they have revealed barriers that may hinder the widespread adoption of these devices within this setting. A recent scoping review examining the use of touchscreen tablets with people with dementia in care settings (Hung et al., 2021) identified a range of barriers including care staff’s limited knowledge of the devices – which could detrimentally impact their willingness to use them; a lack of WiFi or internet connection within care homes; and the physical accessibility of the devices, with factors such as the weight of the tablets and the reflective and/or overly sensitive screens making it difficult for some people with dementia to interact with them. Other research has suggested the cost of iPads may be prohibitive for care homes (Evans, Bray and Evans, 2017).

There is a dearth of studies exploring the use of the Nintendo Wii or Microsoft Kinect in care homes with people with dementia. Those that exist highlight challenges concerning staff and/or volunteers having limited time to set up the technology or deliver the activities (Gerling, Mandryk and Linehan, 2015) as well as the complex gaming mechanisms, particularly with the Nintendo Wii (pushing buttons whilst physically undertaking a range of movements) that can be difficult for older people to learn, especially if they have cognitive and/or mobility impairments (Marston, Greenlay and van Hoof, 2013).

Care home practitioners (including those who provide direct care and support for residents with dementia as well as managers who are responsible for care planning, scheduling and budgeting) are likely to have an important role in incorporating gaming technology more widely into care homes. Consequently, there is a need to explore their perceptions of the challenges and facilitators for its adoption. As these perceptions have previously not been examined, the present study is the first to address this gap in the literature. Furthermore, it is important to situate and interpret this research within a framework that can provide a theoretical interpretation of the current individual and organisatational level barriers that may be impeding the uptake of digital gaming technology within care homes. Elucidating these will enable theoretically driven recommendations to be made for potential behaviour change interventions that will target the appropriate mechanism to support care home staff to use these devices within their care practice. This in turn may begin to facilitate a culture change within care homes, where gaming technology is regularly promoted and used to provide stimulating and enjoyable activities that are beneficial for the well-being of people with dementia.

**Figure 1.**
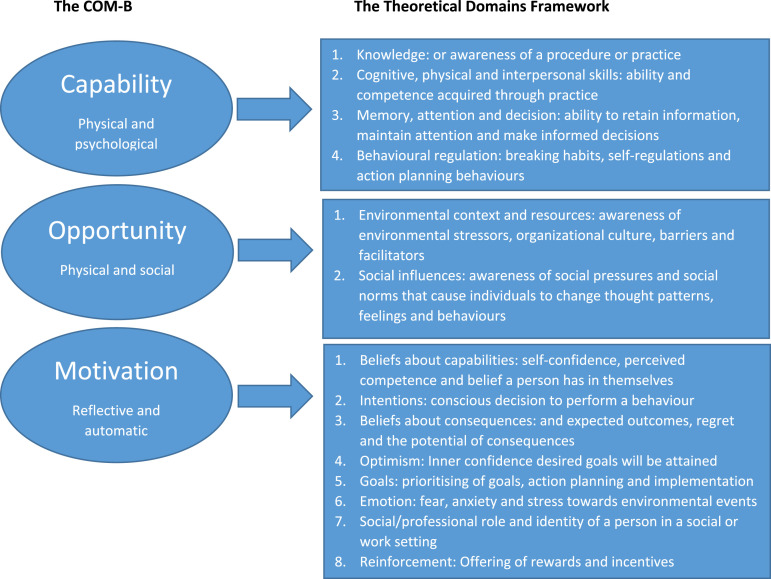
Combining the Capability, Opportunity, Motivation and Behaviour model (COM-B) with the Theoretical Domains Frameowrk (adapted from De Leo, A., Bayes, S., Bloxsome, D. and Butt, J. (2021). The COM-B and The Theoretical Domains Framework.

To achieve this secondary aim, this study drew upon a combination of the Capability, Opportunity, Motivation and Behaviour model and the Theoretical Domains Framework. Combining these theoretical models has been advocated and undertaken within dementia care (Ayton et al., 2020) and dementia education (Surr et al., 2020) interventions as they enable a detailed exploration of implementation barriers at an individual and organisational level; something that the current research seeks to address. The Capability, Opportunity, Motivation and Behaviour model is widely used to contextualise individual-level change and the underlying determinants to achieve organisational change (Michie, van Stralen and West, 2011). It seeks to establish how capabilities (a person’s knowledge and skills), opportunity (social, interpersonal and environmental factors that mediate certain behaviours) and motivation (individual cognitive and emotional processes that direct behaviour) can be used to understand an individual’s behaviour. These three domains are further sub-divided into six sub-domains that outline the factors influencing a person’s individual capacity to adopt new behaviours. The Theoretical Domains Framework builds on the systems identified in the Capability, Opportunity, Motivation and Behaviour model to provide 14 domains to categorise the potential behavioural and organisational factors that can influence implementation outcomes (Atkins et al., 2017). It provides a theoretical lens through which to view the cognitive, affective, social and environmental influences on behaviour. A matrix mapping the Capability, Opportunity, Motivation and Behaviour model and the Theoretical Domains Framework can be seen in Figure 1. This was used as a lens after the inductive analysis of our findings to provide a comprehensive framework for interpreting our findings and so posit which factors may be important during future intervention design.

## Design and methods

### Research approach

This research sought to explore care practitioners’ perceptions of using digital gaming technology within their practice with the objectives of understanding the barriers and facilitators for its widespread adoption within care homes. An exploratory, descriptive qualitative study was deemed most suitable to achieve these objectives given the dearth of research within this field and the need to better understand care practitioners’ perspectives on this topic. An inductive thematic approach was adopted to ensure the findings could be grounded within the varying perspectives of the participants as well as take into account their social context (Flick, 2014). A combination of Theoretical Domains Frameowrk and the Capability, Opportunity, Motivation and Behaviour model were applied afterwards as an interpretative lens for discussing the findings. Data were collected as part of a wider project that aimed to develop and evaluate an online platform (GamePlan) and face-to-face training programme that equips care staff with the knowledge and practical skills to use digital gaming technology as a means to enhance the well-being of people with dementia.

This paper reports on the first phase of the project, where care home practitioners were as follows: (i) consulted on the objectives of the research; (ii) asked to discuss possible barriers and facilitators for using gaming technology within their workplace; and (iii) asked for views about the look and function of the ‘Gameplan’ platform. This paper focuses on data elicited on technology barriers and facilitators. A second paper is in preparation that will outline the development of the ‘GamePlan’ platform. Ethical approval for the research was granted by Bournemouth University.

### Participant recruitment

The research was advertised through project partners, including University networks and Alive charity (https://aliveactivities.org/). Care homes across the south of England were purposively targeted for recruitment. This involved the following: (1) emailing them a link advertising the research, (2) phoning them to discuss the study and to encourage them to promote it amongst their staff and (3) where possible, visiting care homes to distribute advertisement flyers to care staff. Interested participants were asked to contact the project manager and were then provided further information about the study.

Potential participants were provided with the information sheet approximately 1 week prior to the focus group to allow them enough time to digest the information and make an informed decision on whether to take part. If they worked with people with dementia and were interested in promoting the use of gaming technology in care homes, they were asked to register their interest in participating. A total of 54 participants signed up, although a final convenience sample of 48 practitioners took part in the research. Participation was voluntary with no monetary incentives provided.

Although the majority of attendees worked within care homes, it was acknowledged that the project may be of interest and applicable to other dementia practitioners such as those working in Day Centres, and so their insights were welcomed. Whilst data collected from participants working outside of the care home sector were useful for informing the development of the ‘GamePlan’ platform and providing an overview of gaming technology use within the wider dementia care arena, for the purposes of this paper, only data elicited from the 39 care home practitioners are reported to ensure the findings can be situated within this social context. These participants included the following: ‘*care workers’* who provided daily care and support to residents with dementia; *‘Activity Co-ordinators’* who were responsible for planning and delivering psychosocial activities for care home residents; and *‘care home managers’* who were responsible for managing the scheduling and budgeting of activities. Primarily, participants worked within a private care setting. The demographic characteristics of the participants are presented in Table 1.

**Table 1.** Participant demographics.

FG	Venue	N	Gender	Age	Role	Years in care employment
1	Care home	7	M= 1 F= 6	26–34 = 1 35–44 = 1 45–54 = 3 55+ = 2	Care worker = 4 Activity Co-ordinator = 1 Care home manager = 2	0–5= 1 6–10 = 2 11–20 = 2 21+ = 2
2	Care home	7	F = 7	35–44 = 3 45–54 = 3 55+ = 1	Care worker = 3 Activity Co-ordinator = 1 Care home manager = 3	0–5 = 3 6–10 = 2 21+ = 2
3	Care home	6	M = 2 F = 4	18–25 = 2 6–34 = 1 35–44 = 2 55+ = 1	Care worker = 4 Activity Co-ordinator = 2	0–5 = 4 6–10 = 1 11–20 = 1
4	University	2	F = 2	26–34 = 2	Care worker = 2	0–5 = 2
5	Community Centre	8	F = 8	18–25 = 2 26–34 = 1 35–44 = 1 45– 54 = 2 55+ = 2	Care worker = 2 Activity Co-ordinator = 6	0–5 = 4 6–10 = 1 11–20 = 2 DNR = 1
6	Community Centre	2	F= 2	45–54 = 1 55+ = 1	Activity Co-ordinator= 1 Care home manager = 1	0–5 = 1 21+ = 1
7	University	2	M= 1 F = 1	26–34 = 1 35–44 = 1	Care worker = 1 Care home manager = 1	0–5 = 1 6–10 = 1
8	Care home	5	F= 5	35–44= 2 45–54 = 2 55+= 1	Care worker = 1 Activity Co-ordinator = 4	0–5 = 2 6–10 = 1 21+ = 2

### Focus groups

Focus groups were deemed the most appropriate method to incorporate a wide variety of opinions and enable a dynamic dialogue and interaction, thereby ensuring the collection of rich data. The method also enabled a degree of quality control on data collection, as participants were able to provide checks to eliminate false or extreme views (Flick, 2014). Eight face-to-face focus groups were conducted across the south of England between November 2017–February 2018. They took place in a range of settings including care homes, community centres and the university. Each focus group ranged in duration from 105–125 min excluding consent processes. Written consent from the participants was obtained at the start of the focus group. Although the participants had received the information sheet prior to attending, a second copy was provided to them on the day and the lead author (BH) gave an overview of the study aims and objectives. During this process the participants had the opportunity to ask any questions regarding their participation in the study and the use of their data. After eight focus groups it was evident that data saturation had been reached and no new insights were emerging.

The focus groups were conducted by the lead author (BH) who has a PhD and extensive experience undertaking qualitative research. He was supported by a post-doctoral researcher and a member of the Alive team (MB). Where numbers permitted (see Table 1; focus groups 1, 2, 3 and 5), participants were split into smaller groups within the focus groups to share their experiences. These discussions were facilitated by the research team members who then fed back the key points as part of a wider group conversation. This ensured participants had time and space to voice their opinions, and by regularly alternating the members in each of the small groups it enabled them to develop a level of rapport during the consultation. Although in some focus groups more than one care practitioner from the same workplace attended, in the majority of cases the participants were unknown to one another.

**Table 2.** Focus group schedule.

1	Perceptions of Off-the-shelf digital gaming technology
	What do you understand by gaming technology?
	What sort of devices are you aware of?
	What are your thoughts on using gaming technology with people with dementia?
2	Experiences of using gaming technology in care practice
	Can you discuss any experiences you have had of using gaming technology?
	Any experiences of using gaming technology within the care home and with people with dementia?
	How did you find this?
	Did you experience any challenges?
	What were these?
	How did you look to overcome them?
	Would you use gaming technology again with people with dementia?
	For those who have not used it within their care practice why might this be?
	Would anything help you to consider using it within your practice?
3	Thinking more generally now about your care home/place of work, what do you think may be the barriers to using gaming technology within dementia care practice?
	(can prompt on barriers faced by colleagues, organisational barriers, barriers associated with people with dementia)
4	What do you think may encourage/support your care home to adopt the use of gaming technology with people with dementia more widely?

A semi-structured focus group schedule was used flexibly and the questions aimed to elicit participants’ perceptions and experiences of using digital gaming technology with people with dementia and the barriers and facilitators for incorporating it within their workplace (see Table 2). Prompts were used throughout the discussions to gather more detailed data on participants’ experiences and perceptions, and to ascertain whether other members of the group were in agreement with the views put forward. These included the following: ‘could you elaborate on that please’? ‘why do you say that’? ‘could you give me an example of that please’? or ‘what do others think about this suggestion’?

All focus groups were audio recorded using a digital dictaphone and following completion they were transcribed, anonymised and uploaded to NVivo 12 to manage the data analysis process. Pseudonyms preserved individuals’ identities.

### Researchers’ reflexivity

All researchers involved in the consultation sessions had previous experience of successfully using gaming technology with people with dementia, and as such were advocates of this medium within the dementia care field. This may have influenced the way the consultation sessions were delivered and consequently the final data obtained. Equally, the participants were predominantly open to the use of technology within dementia care and keen to develop their skills with this medium. This is likely to have created a pro-technology environment during data collection that may have influenced the research outcomes.

### Data analysis

A six-phase inductive thematic approach (Braun & Clarke, 2006) was adopted to analyse the data, which was undertaken by BH, AK, EJ and the Post-Doctoral researcher. Working as a team of four researchers during the analysis mitigated some of the issues associated with researcher bias, particularly as AK and EJ were not involved in data collection and so retained a level of objectivity. A detailed overview of the analysis process is presented in Table 3.

**Table 3.** Inductive analysis process based on Braun and Clarke (2006).

Six-phase thematic approach	Actions	Approach
1. Data familiarisation	The transcripts were read and re-read by BH, AK, EJ and the Post-Doctoral researcher to familiarise themselves with the data.	Analysis was conducted inductively to allow the themes constructed from the data to be grounded within the words of the participants and their experiences.
2. Initial codes developed	Data were coded separately by all researchers.individual transcripts were read line by line to identify key messages into codes. This occurred at a semantic and latent level. Regular meetings occurred between the research team to discuss the codes and draw up definitions for each one that could then be applied by each researcher to the transcripts. This process was overseen by BH and ensured consistency in the analysis process. Any discrepancies between researchers in the coding of segments of text were discussed as a collective and an agreement reached	
3. Themes searched	Relevant codes were collated into potential themes through discussions between the research team. Codes that were not deemed relevant were recorded as miscellaneous.	
4. Themes reviewed	Thematic mind-maps were developed to better understand how the themes sat together and to construct higher level themes. These were discussed among BH, AK, EJ and the Post-Doctoral researcher.	
5. Themes defined and named	Themes were reviewed by the wider research team (CC, WT, ST, SN) and then defined and named. Miscellaneous codes were re-visited to examine whether they sat within the wider findings. A cross case analysis was undertaken to explore differences in themes between focus groups.	
6. Write-up	The most appropriate participant quotes illustrating the key points pertinent to the research were selected for the final manuscript. The inductive themes and codes were then considered through the lens of the Capability, Opportunity, Motivation and Behaviour model and Theoretical Domains Framework to characterise individual, social and organisational barriers to adoption of gaming technology in care home settings.	

## Findings

Three key themes were constructed from the focus group data highlighting the *care environment, knowledge and skills for inclusive gaming, and staff attitudes and beliefs about capabilities* as factors perceived to hinder and facilitate the uptake of gaming technology in care homes. These themes, along with their respective sub-themes, are discussed below. All supporting quotes have been outlined in Table 4 and referenced within the themes.

## Care environment

Across all focus groups, care practitioners highlighted how aspects of the care environment hinder the adoption of gaming technology and offered suggestions on how these challenges may be overcome.

### 24/7 culture of care

The majority of care practitioners discussed their high workload and the *‘24/7 level of care’ (FG5, P1)* they were expected to provide to the residents in their care homes. With limited time, care practitioners felt that the emphasis of their job role was to provide care for their residents and ensure that their basic needs were met; such as being clean and fed and up to date with medication (Q1).

This resulted in little focus or time being allocated to providing mental, physical or social stimulation despite an understanding from care practitioners that these facets are equally important for residents’ well-being (Q2).

Some participants noted that this culture could sometimes be perpetuated by managers who prioritise the care of residents over their need for stimulation, and consequently some care workers felt that providing residents with activities was beyond the remit of their job and more applicable to other staff such as Activity Co-ordinators (Q3). Constraints on care staff time also meant that when technology was introduced into the care home setting they were unable to learn how to use it or *‘invest and find all the things that you need on it’ (FG1, P5).* With limited incentivisation from managers this could mean that it was often left untouched.

Some care homes had the resources to employ Activity Co-ordinators and participants discussed how it had been beneficial to have someone with the time and motivation to explore, plan and implement new activities for their residents. Other practitioners highlighted how their care home did not have the resources to employ Activity Co-ordinators and instead encouraged volunteers to deliver activities in the homes; acknowledging that the success of this depended on the volunteers’ skills and how much time they could commit each week. Although these offered solutions that supported residents to remain stimulated, participants suggested there was a need for culture change in care homes so that care staff saw it as part of their remit to provide activities alongside care. They felt that gaming technology was something that could be easily integrated into everyday care practices to provide stimulation for residents, without overly increasing demands on care staff (Q4).

### Technological infrastructure

The participants discussed the importance of ensuring care homes had the infrastructure to enable these technological devices to be used to their full capacity. This included appropriate and accessible rooms for running the activities as well as internet availability across the care setting. When discussing the accessibility of the rooms, participants highlighted the need for rooms that were spacious enough to allow people with dementia to move around freely and safely when engaging with sensory gaming technology such as the Nintendo Wii or Microsoft Kinect. These rooms would also need to accommodate larger TV screens that would enable standing/sitting from an appropriate distance, group activity, and/or to accommodate those with visual impairments. In addition to this, participants felt it was important to have rooms that offered a level of privacy, particularly for one-to-one or small group activities where people were sharing potentially personal information (Q5).

Participants noted that while it may be possible for larger care homes to allocate specific rooms to certain tech-related activities, this may not be feasible in smaller care homes with limited communal rooms and/or space. Furthermore, some participants discussed the importance of having reliable and strong internet coverage throughout the care home. This ensures gaming technology such as tablets, which often require WiFi to download and run applications, could operate to their full capacity. They discussed areas within their care home, even in communal rooms for residents, where there was limited or ‘sporadic’ (FG2, P7) internet signal and this would restrict the use of these technologies within these areas (Q6).

To address these issues participants suggested WiFi boosters could be used to amplify the signal throughout the care home or activities could be downloaded onto tablets before delivering them. However, they acknowledged that this latter suggestion was not ideal as it required staff to plan ahead and have a detailed understanding of their residents’ interests and hobbies before delivering the activities, thereby preventing them from responding in the moment as these became known.

### Frugal management

Many participants across all focus groups discussed how the care sector was operating within times of fiscal restraint and so many of the decisions being made *‘come down to money really for most times’*. *(FG7, P1)* (Q7)*.*

Consequently, they highlighted how many managers were working with restricted budgets and this could impact their willingness to prioritise the purchase of gaming technology and the associated games and/or applications, particularly if they believed only a few of their residents may engage with them. This issue was exacerbated when managers and care staff were not technology-orientated and perceived the technology as expensive and/or were unsure of the most appropriate devices to buy for their residents. Often this led to them refraining from purchasing any equipment for fear of wasting limited funding resources (Q8).

To address these issues, participants highlighted the need to develop a cost-benefit case for purchasing these technological devices that they could present to managers. This required them to draw on a wide range of information to demonstrate the variety of activities available through the technology, how the devices could be used to appeal to and benefit the well-being of residents as well as where they could be purchased at reduced cost (such as from websites selling second-hand devices) (Q9).

However, they acknowledged that creating a business case could be time-consuming and the evidence was not always accessible to them, particularly if it was published in academic journals that were not open access. This could make it difficult for them to present a strong argument for purchasing gaming technology. Where budget restrictions had prevented care homes from buying devices, care practitioners discussed the possibility of holding fundraisers or applying for grants from local and national charities.

## Knowledge and skills for inclusive gaming

This theme concerns the importance of care staff having knowledge of gaming technology and their residents’ interests and capabilities, as well as the necessary skills to manage ongoing engagement and well-being during the activities. Across all focus groups, participants noted that technology in general, as well as gaming technology specifically, was not widely adopted within the care home sector, and particularly amongst those from an older generation (Q10). This prevented them from understanding what devices were available and how to use them inclusively with people with dementia. Participants who had experience of using technology within their care practice were able to draw on these insights to highlight the interactional barriers that could arise if care staff were inexperienced with gaming technology and/or unfamiliar with the resident they using it with. They were also able, through experience, to strategic solutions to these barriers.

### Managing engagement of the learner

Participants emphasised that whilst it was possible for people with dementia to engage with gaming technology there was still a need to recognise that these were commercially produced devices and so *‘none of these will have been built with dementia in mind’* (FG7, P2). Consequently, there were inevitable challenges that needed to be managed appropriately by staff when selecting and introducing gaming technology to this population. Participants highlighted how people with dementia may have an initial apprehension of these devices, particularly if they have never used it before, and this can result in a reluctance to engage with it when first introduced. Consequently care staff needed to be mindful not to overwhelm people with dementia during these introductory stages. As one participant stated: *‘If they don’t want to use technology, we don’t want to scare them’ (FG3, P1).* Participants suggested that care staff who are novices to the technology, lacking the knowledge to select the appropriate devices and the skills to introduce it in a considerate manner, may find this challenging. This could lead to a reluctance to engage people with dementia with gaming technology.

Furthermore, participants discussed how it can be hard to maintain the engagement of people with dementia during a technology session and that certain games and activities may be difficult for people to interact with depending on their cognitive and physical abilities (sight, hearing, etc.) as well as the complexities of the gaming mechanisms. Again, these challenges may act as barriers for care staff if they are unaccustomed to using gaming technology and so do not have the knowledge and skills to modify the devices or games so they better align with the capabilities of the person with dementia (Q11).

To overcome these barriers, participants discussed the need for staff to have a sound understanding of each device and the available games to enable them to select the most appropriate ones for their residents and to adjust the difficulty settings to ensure greater levels of engagement and more positive outcomes. They also emphasised the need for staff to remember and adopt the person-centred approaches they have been taught throughout their care career and appreciate the individuality of their residents. Individuality included residents’ interests, hobbies and skillsets as well as the times of the day when they function at their best (such as in the morning or after their pain medication) (Q12).

Furthermore, participants highlighted the need for care staff to demonstrate high levels of softer, personal skills (being calm, patient and attentive) and good communication abilities when introducing and using the technology with people with dementia. They also emphasised the need to create a positive and supportive group atmosphere that encourages people to participate even if they are unable to successfully interact with the game. As one participant noted: *‘You’ve got to remember, it’s not about winning, it’s about taking part’! (FG6, P1).*

### Managing the ongoing well-being of the learner

Participants suggested that care staff’s lack of knowledge of gaming technology may present challenges when managing the physical, mental or emotional well-being of people with dementia as they interact with the devices. This may result in them inadvertently causing harm to the person with dementia, which could discourage future engagement with the activities. Again, these challenges are also likely to vary depending on the characteristics and capabilities of the residents; thereby further emphasising the need for care staff to have a good knowledge of the games as well as the resident. As one participant stated: *‘Something that is amazing for one person, may be hell for another’ (FG2, P7*).

The physical challenges concerned the trip hazards that can be present from trailing console cables as well as people overexerting themselves whilst using sensor technologies and then falling or accidently letting go of controllers (such as the Nintendo Wii remote controllers). Furthermore, other participants discussed residents within their care home who were epileptic and may be affected by the flashing graphics within the games.

The mental challenges related to the potential for emotional distress in people with dementia as they interact with the games. This might include people feeling a sense of frustration or agitation if they cannot engage with a game as well as they hoped, thereby *‘identifying their failings to them’ (FG5, P5),* becoming unsettled by the immersive components of a game, or being reminded of sad and potentially traumatic events during reminiscence activities using iPads (Q13).

To address these challenges, participants emphasised the importance of staff undertaking a thorough risk assessment before running any activities as well as familiarising themselves with the games in advance. This enables the identification of potential physical hazards as well as aspects of games that some people with dementia could struggle with or find distressful or unsettling. Where potential challenges could not have been foreseen, such as people becoming distressed during reminiscence activities, participants highlighted the importance of care staff having the interpersonal skills needed to alleviate the situation.

## Staff perceptions about capabilities

Care staff’s lack of knowledge about gaming technology could also result in negative perceptions towards it as an approporiate medium for engaging people with dementia as well as a lack of confidence in their own abilities to interact with it. This could further contribute to a reluctance to use it within their practice. Participants with experiences of working with care practitioners who held these perceptions were able to offer suggestions on how to overcome these challenges.

**Table 4.** Illustrative quotes to support the final themes.

Higher order theme	Sub-theme	Supporting quotes
Care environment	24/7 care culture	Q1: P1: ‘…there’s no time to get around everyone and actually spend some amount of time with someone’
		P4: ‘that’s what I was thinking’.
		P5: ‘I mean we would be loving to do that, but we haven’t got that time’. (FG2)
		Q2: ‘the physical or the medical and care needs are looked after but the mental and physical activity needs I don’t think are met very often’, (FG4, P1).
		Q3: ‘I think you need to have somebody who has that focus, whose focus is not about the care, whose focus is about mental and physical stimulation’, (FG7, P1).
		Q4: ‘…while you are tidying up in the room, you can give the tablet (to the resident) and do something’, (FG8, P2)
		‘It would it be useful for you… Let’s say…. someone is anxious this morning, put a little music on it (the tablet), you can create an activity out of that’, (FG5, P3).
	Technological infrastructure	Q5: ‘…because you wouldn’t want staff, sort of, Dick, Tom and Harry, sort of, coming in and interrupting, if that was a session you were trying to run’ (FG8, P4).
		Q6: ‘So whereas you might have some people maybe that are in bed more, and you want to take the activity to them, it will depend at the moment what part of the building they are in, as to whether it will work or not’. (FG6, P1)
	Frugal management	Q7: ‘health and social care budgets are shrinking and shrinking all the time, there’s less and less money out there, you know’. (FG4, P2)
		Q8: ‘When you’re not tech-minded you won’t know what’s good or not. You may be wasting money, so I think the initial purchase can be a barrier, because if you get it wrong, you will waste money’. (FG5, P3)
		Q9: ‘So if I go to my manager and say I want to spend £1000 on iPads and a projector and an Apple TV … you know, I have to justify that by saying, “and this is the outcomes that I can achieve with these clients,” because that’s actually what we’re selling ourselves on’. (FG7, P2)
Knowledge and skills of inclusive gaming	Managing engagement of learner	Q10: ‘We’ve got staff that have never had an email address in their life…So it’s difficult for them to get their head around that (gaming technology)’. (FG1, P4).
		Q11: ‘And you find that with obviously the dementia, if people are sitting there for four or five minutes while games are loading or setting up, people can get bored and walk around. So it can be difficult to keep that group going’. (FG6, P2)
		Q12: ‘It’s important that care staff remember that everyone’s an individual. What works for one person may not work for someone else. Some people like touch or sensory, some people like music. So what you need to do is supply all of those possibilities’. (FG3, P3)
	Managing ongoing well-being of learner	Q13: ‘The iPad is fantastic in one respect, for reminiscence and stuff like that but you need to know the person really well. Because if you’re all sitting and reminiscing about something…You’re potentially putting them back in a situation that pulls them into distress’. (FG2, P4)
Staff perceptions about capabilities	Staff attitudes towards technology	Q14: ‘…the biggest problem is care staff, because they’ve got to be passionate about wanting to provide activities, and if they’re not, it doesn’t matter what you give them, they just won’t be bothered’. (FG4, P2)
		Q15: ‘There’s a kind of fear factor associated with using technology…I didn’t come into care to do paperwork or use technology…. And so I think this (using gaming technology) can be a challenge and a daunting one. People are not confident’. (FG2, P4)
		Q16: ‘I do find it in my home that we have carers that come from different countries and their technology is in their own language. And that could be a barrier for some of our staff to try to use these technologies in English as well’. (FG5, P5)
		Q17: ‘I think we have a habit in care - oh it’s something new, and then it doesn’t work the first time, we won’t do it again’. (FG4, P2)
	Staff attitudes towards capabilities of people with dementia	Q18: ‘I think, there’s a broadly held perception (among care staff) that technology is for the young…Older people just aren’t interested in that technology’. (FG1, P4)
		Q19: ‘We mostly have people with dementia (in the care home) and I don’t want to be rude, but they are already in a different world and I think it would be too much stimulation for them’. (FG3, P2)
		Q20: ‘Well also I've noticed a lot of our dementia clients are getting younger. You can't just say they all like listening to war songs…So they would grow up being more technical based than the people that are in their 90s, or 103. So they would probably embrace it a lot better I think’. (FG6, P1)

### Staff attitudes towards the technology

Participants perceived staff perceptions of gaming technology as integral to ensuring it is employed as an activity to enhance the well-being of people with dementia in care homes (Q14). Participants felt a lack of knowledge about the devices among care staff resulted in them feeling uncomfortable and apprehensive around it, and thus reluctant to set it up and use it in their work practice with people with dementia (Q15).

Participants noted that this low self-efficacy may also result in staff resisting opportunities for change within their job role or engaging in new learning that might put them outside of their comfort zone. If a member of staff’s native language was not English, this could create additional challenges for them to engage with shared technology and games in the care home where the language had been pre-set to English (Q16).

Participants highlighted how feelings of incompetence and negative attitudes towards gaming devices, could, in turn, create a defeatist mind-set or *‘put them on a negative footing’* (FG8, P5) prior to using them with people with dementia. In such instances, if they were to engage with gaming technology and the activities did not pan out as planned, care staff were less likely to persevere with them and merely accept the outcome as confirmation of their initial misgivings and so refuse to engage with them again (Q17).

To address these challenges, participants emphasised the importance of providing staff with training, time and opportunities in their working day to become familiar with gaming technologies and learn how to set them up and use them. It was envisaged that increasing confidence, familiarity and competence with the devices could shift pre-existing views and encourage adoption. These attributes of confidence, positivity and persistence will be particularly important when working with a population who themselves are likely to be unfamiliar and inexperienced with gaming technology and so apprehensive towards engaging with it.

### Staff perceptions of the capabilities of people with dementia

Participants also suggested that some staff held misapprehensions regarding the interests and abilities of people with dementia – and older people more generally – to engage in activities using these devices (Q18).

Although the participants felt that many of the care staff were well-trained in general dementia awareness and education, it was felt that some assumed that people with dementia were unable to *‘pick up new things’ (FG8, P1)* or engage with a virtual environment. This belief that they would be incapable of interacting with GT, particularly if they had not used it prior to the onset of dementia, could lead to a defeatist mind-set on the part of the care practitioners and so reinforce a reluctance to use gaming technology with people with dementia (Q19).

However, some participants acknowledged that their younger residents with dementia were likely to be more technologically savvy and willing to engage with it. They noted that the demographics of their residents are shifting with some now more likely to have engaged with technology previously and so be keen to continue to do so. As such, it was the responsibility of care staff to *‘keep up with the times’ (FG8, P4)* and provide residents with opportunities to participate in new tech-related activities (Q20).

To address these challenges, participants highlighted the need to promote learning that incorporates demonstrations of people with dementia successfully engaging with gaming technology and provides examples of the benefits this can have for their well-being. They felt that if this learning was communicated and championed through their peers then this may carry more weight and care staff may be more likely to listen and make efforts to adopt it within their practice. Furthermore, they emphasised a need for care staff to keep an open mind and refrain from pre-judging whether a person with dementia will be able to engage with the technology, as for some, cognitive and physical abilities may fluctuate, even on a daily basis.

## Discussion

This is the first study that has explored the perceptions of care home practitioners on the potential challenges and facilitators for the widespread adoption of gaming technology within their work practice. Our findings, constructed from an inductive analysis of the data, suggested that practitioners were able to provide detailed insights into these issues.

**Figure 2.**
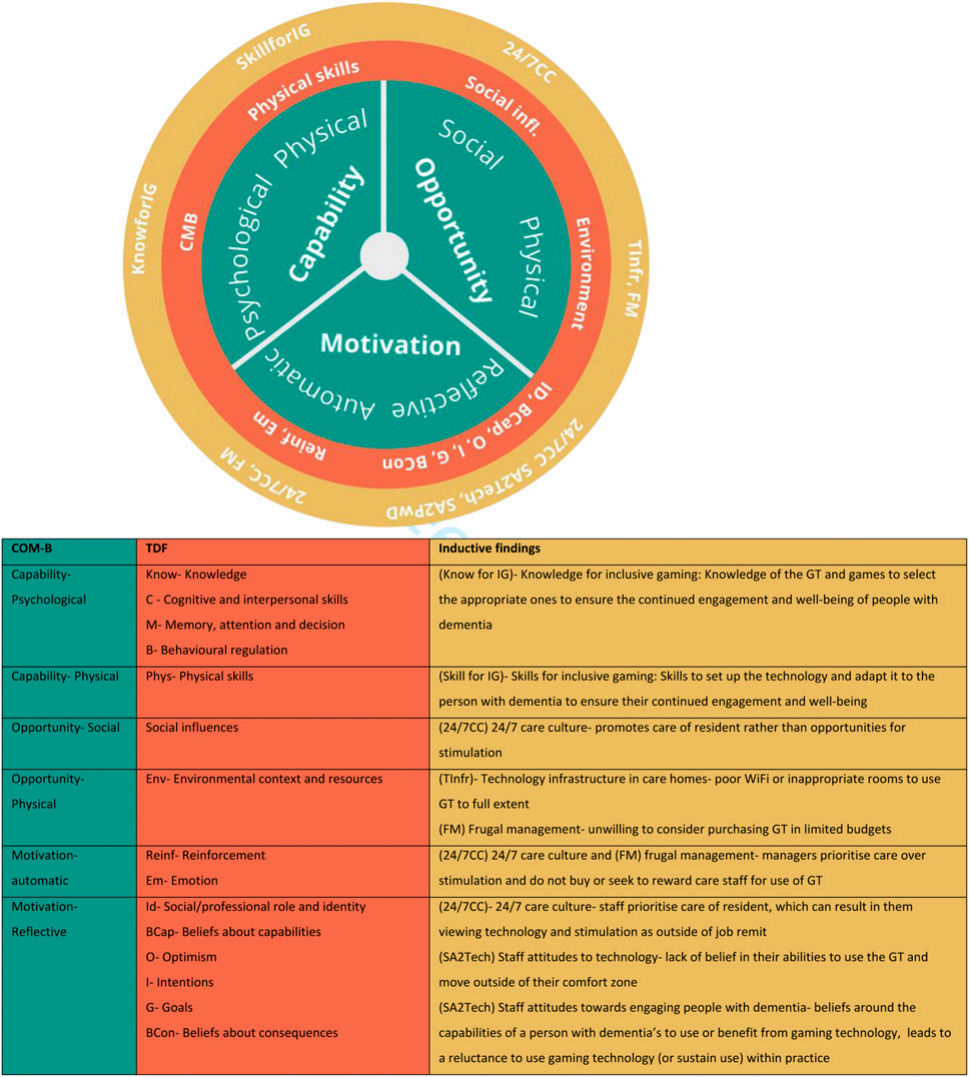
Our inductive findings mapped onto the combined and TDF model. (adapted from Atkins et al. (2011)).

Interpreting these through a combined lens of the Capability, Opportunity, Motivation and Behaviour model and the Theoretical Domains Framework provides a structured, theory-based understanding of why this technology is yet to be widely adopted within the care sector as well as informing how these challenges can be overcome. As illustrated in Figure 2, examining the three key themes through these combined frameworks emphasises how they work together to inhibit the capabilities, opportunities and motivation for care staff to use gaming technology in their practice. Consequently, facilitators must be targeted at all levels to bring about culture change within the care home sector.

### Capability for using off-the-shelf gaming technology

The Capability, Opportunity, Motivation and Behaviour model and Theoretical Domains Framework outline the importance of ensuring people have the necessary cognitive and physical skills so that they feel capable of changing their behaviour. As evidenced by our participants, and in accordance with the wider academic literature (Hung et al., 2021), these skills are often lacking in the majority of care staff who rarely engage with gaming technology in their work or general lives. Consequently, as our findings suggest, care practitioners report that they have limited knowledge of what gaming technology is available as well as how to set it up and use it inclusively with people with dementia. This emphasises the need for training that begins at a basic level; outlining the gaming technologies and games available as well as how to set them up and use them in a way that enables people with dementia to engage with them. As proposed by our ‘GamePlan’ project, this training should be developed in collaboration with care home practitioners to ensure that it is fit for purpose and takes into account their social context. For instance, as highlighted by our participants, some care staff may not be native to the UK and so have limited English. Consequently, it is important that the training is provided in a range of mediums such as videos and diagrams to ensure that it is inclusive to the whole care home sector.

The theoretical framework also outlines the importance of providing people with the interpersonal skills as well as the knowledge and physical skills to bring about behaviour change. Within the care home sector, these softer interpersonal skills form the basis of all staff training for working with people with dementia and often focus on the concept of ‘person-centred’ care approaches. These emphasise the need to focus holistically on the person and not their dementia and to consider their life histories, personalities, capabilities and choices to ensure the most appropriate level of care can be provided (Kitwood, 1997). Consequently, situating any training within the framework of ‘person-centred care’ is likely to ensure it is familiar to care staff and also demonstrates how gaming technology can be used to support and enhance these care approaches.

### Opportunity for using off-the-shelf gaming technology

The Capability, Opportunity, Motivation and Behaviour model and Theoretical Domains Framework outline the need for an environmental context and social influences that provide opportunities for people to change their behaviours. Currently, the 24/7 care culture that prioritises the physical care needs of the residents over their mental and social stimulation is unconducive to bringing about the attitudinal and behavioural changes in staff. As highlighted by our participants, if managers do not understand or value the potential benefits that gaming technology offers their residents, they may be unwilling to allocate the necessary, often minimal, resources required to purchase and use it. This finding concurs with other care home research that highlights stimulation for residents is often not prioritised or acknowledged when considering their care needs (Tak et al., 2015). Our findings reaffirm the need to bring about a wider culture change within the care sector. This is likely to be achieved by targeting training not only with care staff but also managers and care home designers to raise awareness of the potential benefits of gaming technology for people with dementia and the importance of allocating resources and budget for it. While, as highlighted by our participants and other research (Evans, Bray and Evans, 2017; Chu et al., 2021), there are workaround solutions for promoting technology use in already established care homes (e.g. WiFi boosters), this training may be particularly beneficial at the early stages of care home development. At this point, planners can design and designate appropriate rooms for group gaming activities and personal one-to-one sessions as well as ensure all rooms can be connected to the WiFi, thereby creating an appropriate physical environment for these activities. This latter point is particularly salient given some of our participants noted a lack of WiFi connectivity within areas of their care home, which served as a barrier to using gaming technology to its full capability. These challenges will be pertinent to address for the next generation of care home residents who are likely to be familiar with these devices and so will expect to be able to access them throughout the care home.

Participants highlighted that in some care homes, Activity Co-ordinators and volunteers provided activities using gaming technology and this was beneficial for the residents’ well-being. However, for long-term culture change within the care home sector, this approach may not be beneficial. The Capability, Opportunity, Motivation and Behaviour model and Theoretical Domains Framework emphasise the need for appropriate social influences to create opportunities for behaviour change. If care home staff only ever see those in other roles undertaking these activities, it is likely to perpetuate the misperception that it is outside their job remit, which our participants suggested was a common assumption among colleagues. To address this, it is imperative that care staff witness their peers using these gaming technologies and promoting their benefits in day-to-day activities with residents, and that this is encouraged by their managers. As highlighted by the participants, creating Tech Ambassadors among care home practitioners is likely to be the most successful approach for establishing a social context that encourages care staff to use gaming technology within their care practice.

However, within the UK care home context, there is high staff turnover and low pay among care workers and this may result in low motivation and difficulty engaging them in additional work (Kupeli et al., 2018). These high levels of staff turnover are important to consider when attempting to introduce and develop the role of Tech Ambassadors. Further research would be beneficial to understand how this train-the-trainer model could be successfully implemented and how it could benefit the development of care staff. Developing a community of Tech Ambassadors and an online platform for the exchange of ideas alongside an accreditation may be one way to motivate and engage people to undertake this role.

### Motivation for using off-the-shelf gaming technology

The Theoretical Domains Framework highlights the importance of examining the attributes of people such as their intentions, beliefs, emotions and goals that, according to the Capability, Opportunity, Motivation and Behaviour model, will create motivational behaviours to bring about personal change. Our participants outlined that care practitioners’ lack of knowledge around gaming technology could result in apprehension and reluctance to engage and/or persist with it. As suggested by participants and in accordance with other researchers (Weiss et al., 2021), it is likely that enhancing this knowledge and experience will, in turn, improve skills, confidence and self-efficacy in using gaming technology, thereby lessening negative attitudes and emotions towards it. However, to further motivate behaviour change, managers must be willing to promote this agenda by encouraging staff during their one-to-one meetings to view it as part of their job role and to set themselves goals to use it within their care practice. Again, this emphasises the need for managers to be included in the training process and to work in collaboration with them to explore how they can motivate their staff to engage with gaming technology. As highlighted earlier, one way to encourage its promotion and incentivisation by managers, and its potential adoption by care staff could be to frame training within the concept of achieving excellent person-centred care. Research has demonstrated how gaming technology can provide opportunities for people with dementia to re-engage virtually with activities that were central to their identity but which they may no longer be able to participate in now, such as golf, bowling and driving on the Nintendo Wii/Microsoft Kinect (Hicks, Innes and Nyman, 2020). Furthermore, these technologies can be used to better understand life histories, likes and dislikes by supporting reminiscence activities using apps such as Google Earth to virtually re-visit places where they grew up and so provide visual prompts to facilitate deeper conversations (Joddrell and Astell, 2016; Hicks, Innes and Nyman, 2020). A better understanding of residents is likely to improve the care provided to them, which will also benefit their well-being and enhance the reputation of the care home.

Our participants also reported a belief that care staff sometimes perceived people with dementia as incapable of engaging with gaming technology and this further added to a reluctance to use it or persist with it, if it was not initially successful (in their eyes). As Genoe (2010) posits, certain activities can often become viewed as ‘dementia-appropriate’ and favoured in the care sector. This can come at the expense of other activities that are then perceived as inappropriate or too difficult to undertake with people with dementia, which further enhances false assertions about the capabilities of this population. To challenge these views, and support care staff motivation, it is likely to be beneficial to create a platform whereby care staff can communicate with and witness their peers successfully using gaming technology in their care practice. Other research has highlighted how engaging people with dementia with these devices can challenge informal carers’ assumptions about the capabilities of this population (Hicks, Innes and Nyman 2020). Therefore, finding a way to promote these positive stories across the care home sector is likely to help with bringing about the necessary culture change.

### Strengths and limitations of the research, and areas for future research

This is the first study to examine care home practitioners’ perceptions of the barriers and facilitators for gaming technology in their care practice, and to examine the findings using a theoretical framework. This has been important for understanding how it may be possible to develop interventions that draw on the appropriate mechanisms to bring about wider behavioural and cultural change within the care home sector. Moving forward, using the combined Capability, Opportunity, Motivation and Behaviour model and the Theoretical Domains Framework it may also be possible for care home managers to identify where barriers for culture change currently exist within their care home and how to address these. As gaming technology continues to advance this is likely to have implications for the costs and utility of devices, which will in turn impact on the potential barriers and facilitators for its use in care homes (e.g. older devices become more affordable and so costs are not as prohibitive to this sector). It is important that researchers and practitioners/care home managers continue to monitor and update their understandings within this field so that sustained behaviour change can be achieved. The Theoretical Domains Framework and Capability, Opportunity, Motivation and Behaviour model provide a useful theoretical framework to facilitate this ongoing process.

It is important to acknowledge that this study drew on the perceptions and reflections of care practitioners. Consequently, we are unable to determine if introducing these suggested measures will overcome the challenges that were highlighted. For a more detailed understanding of these aspects as well as how behavioural and cultural changes can be sustained within care homes, it is likely to be more beneficial to undertake ethnographic or Participatory Action Research. These approaches will enable the theoretical frameworks to be used as a way to identify barriers and introduce, monitor and evaluate measures to overcome these. A further limitation of our work is that it drew on a convenience sample of participants from the south of England, predominantly working in private care homes, who had an interest in gaming technology even if they were not using it. Whilst this approach was necessary given project time and budgetary constraints, caution must be applied when extrapolating the findings to the wider population and those working in state-funded care settings. Further research is required that involves people who may have less interest in, or enthusiasm for technology as well as people working across care homes in other geographical settings with varying resident characteristics to ascertain whether the reported barriers and facilitators are similar.

## Conclusion

This qualitative study, which drew on an inductive thematic approach, explored care home staff perceptions of the barriers and facilitators of using gaming technology within their care practice. Care staff were able to provide detailed insights into these aspects. Examining these within the Capability, Opportunity, Motivation and Behaviour model and Theoretical Domains Framework enabled a more nuanced understanding of the training required and the mechanisms to target in order to create the opportunity, capability and motivation to support behaviour change in care staff and encourage them to engage their residents with dementia with gaming technology. These findings are relevant for current dementia care practice but will also be pertinent for care homes to consider as new technologies emerge within this ever-evolving landscape.

Implications for practice to bring about culture change:

• Incorporation of all stakeholders, including care home designers and care managers in training that outlines the benefits of gaming technology for people with dementia. This will encourage them to consider this during care home design, budgeting and staff development meetings, thereby creating the appropriate environmental context to facilitate gaming technology use among care staff.
• Creation of Tech Ambassadors within care homes to promote the benefits of gaming technology with people with dementia and to work closely with care staff to incorporate it within their care practice.
• Development of accessible training to upskill care staff with knowledge about gaming technology availability and how it can be used inclusively with people with dementia. The training should be based on person-centred care approaches to emphasise how these devices can better support well-established dementia care practices.
• Publication of accessible media highlighting people with dementia successfully engaging with gaming technology to challenge negative pre-conceptions about their capabilities. This should also include content on how the devices and games can be adapted and tailored towards the interests and capabilities of people with dementia.
• Creation of a simple one-stop-shop for information, tips, advice and peer support on using gaming technology with people with dementia. This would need to be designed with capacity to evolve alongside the ever-changing technological landscape.
